# Quality of life after cryoballoon vs. radiofrequency ablation in atrial fibrillation: a subanalysis of the FREEZE cohort study

**DOI:** 10.3389/fcvm.2026.1877743

**Published:** 2026-10-09

**Authors:** J. Pongratz, M. Kuniss, L.-Q. Wu, J. Tebbenjohanns, G. Nölker, U. Dorwarth, K.-H. Kuck, J. Senges, E. Hoffmann, F. Straube, for the FREEZE Cohort Study Investigators, Garcia Alberola A.

**Affiliations:** 1 Klinik für Kardiologie und Internistische Intensivmedizin, München Klinik Bogenhausen, München, Germany; 2 Abteilung für Kardiologie, Kerckhoff Klinik GmbH, Bad Nauheim, Germany; 3 Department of Cardiology, Shanghai Rui Jin Hospital, Shanghai, China; 4 Med. Klinik I, Helios Klinikum Hildesheim GmbH, Hildesheim, Germany; 5 Innere Klinik II/Kardiologie, Christliches Klinikum Unna-Mitte, Unna, Germany; 6 Klinik für Rhythmologie, Universitätsklinikum Schleswig-Holstein, Lübeck, Germany; 7 Stiftung Institut für Herzinfarktforschung, Ludwigshafen, Germany; 8 Ludwig-Maximilians-Universität München, Munich, Germany

**Keywords:** atrial fibrillation, cryoballoon ablation, patient-reported outcome, quality of life, radiofrequency ablation

## Abstract

**Background:**

Catheter ablation for symptomatic atrial fibrillation (AF) is an established treatment, with cryoballoon (CBA) and radiofrequency ablation (RFA) showing similar success rates. However, comparative data on health-related quality of life (HRQoL) outcomes between these methods are limited. This study compares HRQoL changes following CBA vs. RFA in symptomatic AF patients.

**Methods:**

This sub-analysis of the FREEZE Cohort Study (NCT01360008) compared HRQoL outcomes between CBA (group A) and RFA (group B) using the EQ-5D-3L questionnaire and EHRA symptom scores at baseline and follow-up. Changes in mobility, self-care, usual activities, pain/discomfort, anxiety/depression, EQ-VAS, and general physical condition (GPC) were assessed.

**Results:**

Between 2011 and 2016, 4,189 patients were included, with 2,110 (50.4%) completing all HRQoL questionnaires. The mean age was 61.8 ± 10.4 years, with 39.2% female patients. Group A had a higher proportion of patients with paroxysmal AF (72.8% vs. 65.4%, *p* < 0.01). Overall complication rates were similar (7.6% vs. 10.1%, *p* = 0.07). The median follow-up was 434 (375, 517) days. Atrial arrhythmia recurrence was more frequent in group B (45.0% vs. 56.9%, *p* < 0.0001), as were repeat ablations (7.9% vs. 22.3%, *p* < 0.001) and rehospitalizations (26.0% vs. 46.7%, *p* < 0.0001). Both groups experienced significant improvements in HRQoL. After multivariable adjustment, group A remained associated with greater improvement in pain/discomfort (aOR 1.62, 95% CI 1.26–2.08), EQ-VAS (aOR 1.60, 95% CI 1.27–2.02), and the EuroQol 5D-Score (aOR 1.39, 95% CI 1.10–1.76).

**Conclusion:**

Both CBA and RFA significantly improved HRQoL. However, CBA was associated with greater improvements in pain/discomfort, EQ-VAS, and the EuroQol 5D-Score, indicating potential differences in selected HRQoL outcomes between ablation modalities.

**Clinical Trial Registration:** NCT01360008.

## Introduction

Atrial fibrillation (AF), the most common sustained cardiac arrhythmia, presents a significant global health burden, affecting millions of individuals worldwide. Characterized by irregular and often rapid heart rhythms, AF can lead to debilitating symptoms such as palpitations, fatigue, and breathlessness, alongside an increased risk of stroke and heart failure (1). Beyond its physical manifestations, AF profoundly affects health-related quality of life (HRQoL), emphasizing the need for effective treatments that address both clinical outcomes and patient-reported experiences (2). Catheter ablation, an established therapeutic option, has proven effective in reducing arrhythmia recurrence and symptom burden in patients with symptomatic AF. Among the techniques available, cryoballoon ablation (CBA) and radiofrequency ablation (RFA) are widely utilized, with both demonstrating high efficacy in achieving rhythm control and arrhythmia-free survival (1). However, while clinical outcomes such as arrhythmia recurrence and procedural safety are well-documented, the comparative impact of these modalities on HRQoL remains underexplored.

Patient-reported outcomes (PRO) have become increasingly recognized as critical measures of therapeutic success, complementing traditional clinical endpoints (2). Tools such as the EQ-5D-3L questionnaire and European Heart Rhythm Association (EHRA) symptom scores allow for a comprehensive assessment of HRQoL across multiple dimensions, including mobility, pain, self-care, and emotional well-being (3, 4).

Several studies have evaluated the impact of catheter ablation on HRQoL, often demonstrating substantial improvements across physical, psychological, and social domains. For example, randomized trials such as the CABANA study highlighted that catheter ablation significantly enhances HRQoL compared to drug therapy (5). Similarly, studies such as the CASTLE-AF trial and a CIRCA-DOSE substudy have reported significant improvements in functional capacity, symptom burden, and HRQoL following AF ablation. The CASTLE-AF trial specifically demonstrated that ablation in patients with heart failure and reduced ejection fraction led to improved clinical outcomes and HRQoL. A CIRCA-DOSE substudy showed that a reduction in AF burden following ablation was associated with clinically meaningful improvements in HRQoL, with patients experiencing greater benefits when arrhythmia recurrence was prevented (6, 7). However, most prior research has focused on the overall impact of ablation rather than comparing specific techniques like CBA and RFA.

The FREEZE Cluster Cohort Study (NCT01360008) was designed to compare the effectiveness and safety of CBA and RFA in patients with symptomatic paroxysmal or drug-refractory persistent AF. This large, multi-center, real-world study included experienced CBA and RFA centers. The main focus was on atrial arrhythmia recurrence as the primary endpoint, with secondary outcomes including procedural results, safety, and the clinical course. In a substantial subset of participants from the main study, HRQoL was systematically assessed over the course of the study. This sub-analysis offers a unique opportunity to compare CBA and RFA regarding their impact on HRQoL in patients with symptomatic AF, utilizing real-world data from the FREEZE Cohort Study, which reflects the diverse patient population and clinical settings encountered in everyday clinical practice.

## Methods

### Study design

The results from the primary endpoint analysis of the FREEZE Cohort Trial (ClinicalTrials.gov Identifier: NCT01360008), a prospective cluster cohort study, have been published, with detailed methodologies for catheter ablation previously described (8). This sub-analysis specifically focuses on HRQoL differences between CBA (group A) and RFA (group B), an aspect not addressed in prior publications. Inclusion criteria for this sub-analysis were as follows: first-time ablation, paroxysmal or persistent atrial fibrillation lasting less than 1 year, failure of at least one antiarrhythmic drug (AAD), and age ≥18 years. Only patients who completed the requested HRQoL questionnaires before and after the respective ablation procedure were included. The study population selection process is illustrated in Figure 1.

**Figure 1:**
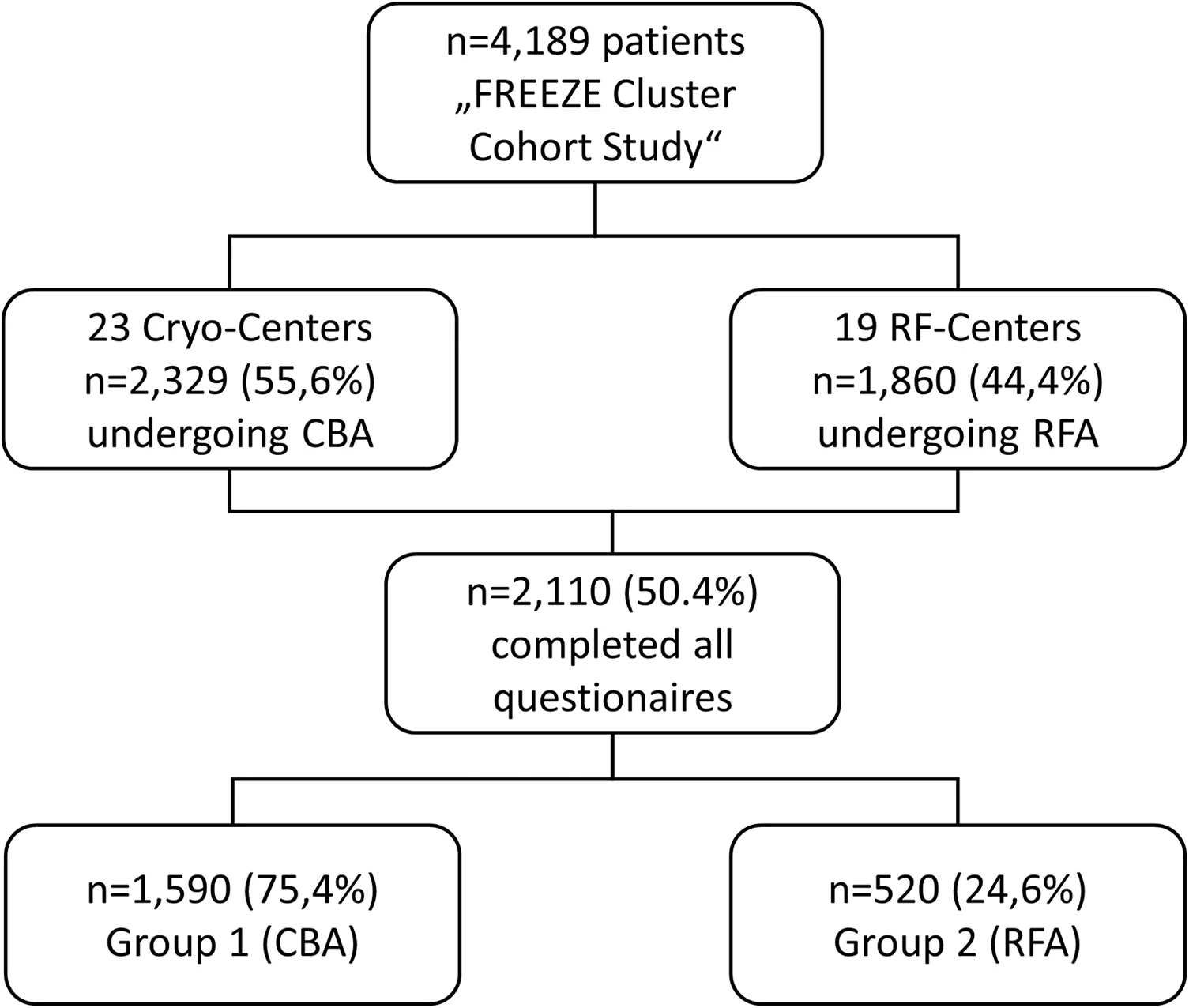
Flowchart of patient enrollment. This flowchart depicts the distribution of patients in the FREEZE Cluster Cohort Study. A total of 4,189 patients were enrolled, with 2,329 patients (55.6%) treated at Cryo-Centers and 1,860 patients (44.4%) treated at RF-Centers. Of the total cohort, 2,110 patients (50.4%) completed all questionnaires. The final sample was divided into two groups: Group 1 (CBA) consisting of 1,590 patients (75.4%) and Group 2 (RFA) consisting of 520 patients (24.6%).

### Quality of life assessment

QoL in this study was assessed using several validated tools: the EQ-5D-3L questionnaire, the EHRA symptom score, and the General Physical Condition (GPC). The EQ-5D-3L is a self-reported measure that evaluates five dimensions of health: mobility, self-care, usual activities, pain/discomfort, and anxiety/depression (9, 10). It provides both a descriptive profile and a summary index, with the EQ-VAS used to assess overall health perception, scored from 0 (worst imaginable health) to 100 (best imaginable health). The EHRA symptom score categorizes symptoms based on severity, ranging from no symptoms (Class I) to severe symptoms that significantly impact daily activities (Class IV). The GPC scale, used to assess general physical condition, evaluates overall health status and physical well-being over the last 12 months. These tools were administered at baseline and follow-up to capture changes in HRQoL and assess the impact of ablation treatments on patients’ daily lives and physical condition. Please refer to Figure 2 for further details.

**Figure 2:**
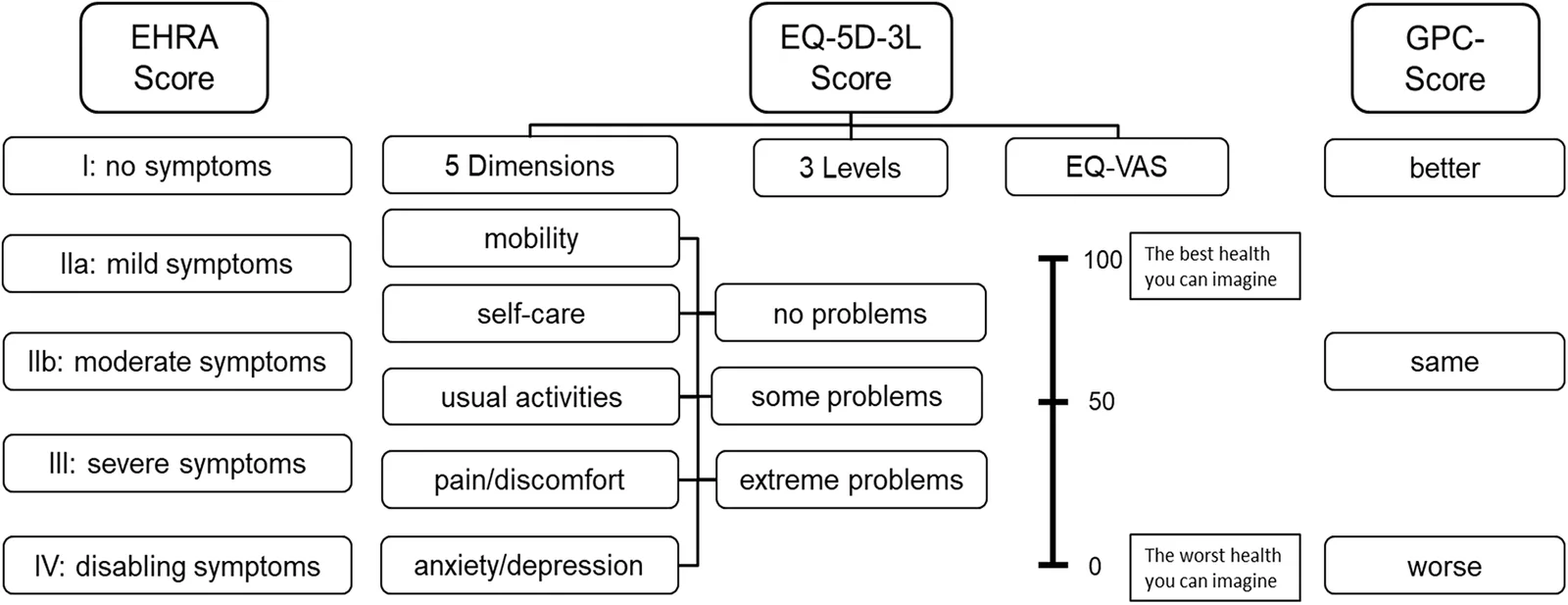
Overview of HRQoL assessment. This figure illustrates the three primary instruments used to assess HRQoL in the study: the EHRA Score, the EQ-5D-3L Score, and the GPC Score. The EHRA Score categorizes symptoms from no symptoms (I) to disabling symptoms (IV). The EQ-5D-3L Score evaluates five dimensions (mobility, self-care, usual activities, pain/discomfort, and anxiety/depression) with three severity levels (no problems, some problems, and extreme problems), and includes the EQ-VAS, which measures overall health on a scale from 0 (worst health) to 100 (best health). The GPC Score reflects overall physical well-being, categorizing it as better, same, or worse during the last 12 months.

### Statistical analysis

Categorical variables were presented as counts and percentages and were compared using the chi-square test. Continuous variables were reported as mean ± standard deviation or median with interquartile range (IQR), as appropriate, and were compared using the Mann–Whitney *U* test. Changes in HRQoL parameters from baseline to follow-up were analyzed and compared between groups.

To account for differences between the treatment groups, additional multivariable regression analyses were performed. Changes in HRQoL categories (better, same, or worse) were analyzed using multinomial logistic regression with a generalized logit link function, with ablation modality (CBA vs. RFA) as the primary predictor. The overall association between ablation modality and HRQoL change was assessed using the Type 3 Wald test. In addition, HRQoL improvement was analyzed as a binary outcome using logistic regression to estimate adjusted odds ratios (aORs) with 95% confidence intervals (CIs). Models were adjusted for age, sex, AF type, baseline EHRA class, baseline EQ-5D/EQ-VAS values, BMI, hypertension, coronary artery disease, chronic kidney disease, CHA_2_DS_2_-VASc score, and follow-up duration. Continuous changes in EQ-VAS and the EuroQol 5D-Score were analyzed using linear regression, and adjusted least-squares mean differences with 95% CIs were estimated.

Analyses of individual HRQoL domains were considered exploratory; therefore, no formal adjustment for multiple testing was applied, and corresponding *p*-values should be interpreted descriptively. All tests were two-tailed, and a *p*-value < 0.05 was considered statistically significant. Statistical analyses were conducted using SAS version 9.4 (Cary, NC, USA).

## Results

### Study population

Between 2011 and 2016, 4,189 patients were enrolled, with 2,110 (50.4%) completing all HRQoL questionnaires. Of these, 1,590 patients were in group A and 520 in group B. The mean age of the cohort was 61.8 ± 10.4 years, with group A averaging 61.4 ± 10.4 years and group B 63.2 ± 10.4 years (*p* < 0.001). The cohort included 39.2% females, with no significant difference between groups (*p* = 0.20). Group B had a higher percentage of persistent AF (27.2% vs. 34.6%, *p* < 0.01) and a higher mean BMI (27.5 ± 4.8 vs. 27.9 ± 4.6, *p* < 0.05). Additionally, group B experienced more frequent episodes of AF (*p* < 0.001). A higher proportion of patients in group A (88.1%) had failed at least one AAD, compared to group B (72.1%, *p* < 0.0001). Group B also had a higher prevalence of comorbidities, including hypertension, coronary artery disease, ≥°II valve disease, ≥°II mitral regurgitation, and chronic kidney disease. The CHA_2_DS_2_-VASc score was significantly higher in group B (2.2 ± 1.4) than in group A (1.8 ± 1.3, *p* < 0.0001). Further baseline characteristics are shown in Table 1.

**Table 1:** Baseline characteristics.

Baseline characteristics	Total	CBA (Group A)	RFA (Group B)	*p*-value
	*n* = 2,110	*n* = 1,590	*n* = 520	
Age, years	61.8 ± 10.4	61.4 ± 10.4	63.2 ± 10.4	<0.001
Females (%)	826 (39.2)	610 (38.4)	216 (41.5)	0.20
BMI kg/m^2^	27.6 ± 4.8, *n* = 2,068	27.5 ± 4.8, *n* = 1,552	27.9 ± 4.6, *n* = 516	<0.05
Persistent AF (%)	613 (29.1)	433 (27.2)	180 (34.6)	<0.01
Inefficacy of at least 1 AAD (%)	1,775 (84.1)	1,400 (88.1)	375 (72.1)	<0.0001
EHRA				<0.0001
I/no (%)	200/2,108 (9.5)	163/1,590 (10.3)	37/518 (7.1)	
II/mild (%)	861/2,108 (40.8)	699/1,590 (44.0)	162/518 (31.3)	
III/severe (%)	986/2,108 (46.8)	688/1,590 (43.3)	298/518 (57.5)	
IV/disabling (%)	61/2,108 (2.9)	40/1,590 (2.5)	21/518 (4.1)	
Frequency of episodes				<0.0001
Occasionally (<1x/month)	295/1,851 (15.9)	240/1,389 (17.3)	55/462 (11.9)	
Intermediate (>1x/month)	1,171/1,851 (63.3)	894/1,389 (64.4)	277/462 (60.0)	
Frequent (daily)	385/1,851 (20.8)	255/1,389 (18.4)	130/462 (28.1)	
Previous device implantation (%)	86/2,110 (4.1)	46/1,590 (2.9)	40/520 (7.7)	<0.0001
Coronary heart disease (%)	229/2,109 (10.9)	152/1,590 (9.6)	77/519 (14.8)	<0.001
Valve disease ≥ °II (%)	109/2,109 (5.2)	64/1,590 (4.0)	45/519 (8.7)	<0.0001
Mitral regurgitation ≥ °II (%)	69/2,105 (3.3)	38/1,586 (2.4)	31/519 (6.0)	<0.0001
LA diameter*, mm	41.0 [38.0, 45.0]	41.0 [38.0, 45.0]	41.0 [38.0, 45.0]	0.25
Ejection fraction* (%)	60.0 [55.0, 64.0]	60.0 [55.0, 65.0]	60.0 [58.0, 62.5]	0.37
CHA_2_DS_2_ VASc Score	1.9 ± 1.4, *n* = 2,107	1.8 ± 1.3, *n* = 1,588	2.2 ± 1.4, *n* = 519	<0.0001
Hypertension (%)	1,323 (62.7)	943 (59.3)	380 (73.1)	<0.0001
CKD (%)	77 (3.6)	40 (2.5)	37 (7.1)	<0.0001
Diabetes mellitus (%)	187 (8.9)	125 (7.9)	62 (11.9)	<0.01
Hypertensive heart disease (%)	499/2,109 (23.7)	387/1,590 (24.3)	112/519 (21.6)	0.20
Cardiomyopathy (%)	67/2,109 (3.2)	51/1,590 (3.2)	16/519 (3.1)	0.89
Prior stroke (%)	58/2,110 (2.7)	47/1,590 (3.0)	11/520 (2.1)	0.31
Structural heart disease (%)	811/2,109 (38.5)	600/1,590 (37.7)	211/519 (40.7)	0.24
Left common ostium (%)	201/1,695 (11.9)	140/1,257 (11.1)	61/438 (13.9)	0.12
Accessory veins (%)	86/1,695 (5.1)	73/1,257 (5.8)	13/438 (3.0)	<0.05

*n* (%), Mean ± SD, or Median (Quartiles) as appropriate according to the test of normal distribution. AF, atrial fibrillation; AAD, antiarrhythmic drug; EHRA, European heart rhythm association; CKD, chronic kidney disease; BMI, body mass index; CBG, cryoballoon generation; LA, left atrium.

*Determined by transthoracic echocardiography.

### Procedural results

Procedural results showed significant differences between groups A and B. Group A underwent fewer total catheters (2.9 ± 1.1 vs. 3.0 ± 1.0, *p* < 0.0001) and left atrial catheters (1.2 ± 0.4 vs. 1.9 ± 0.3, *p* < 0.0001). Left atrial time (95.4 ± 37.9 vs. 139.7 ± 56.4, *p* < 0.0001) and total procedure time (128.3 ± 48.9 vs. 184.8 ± 61.5, *p* < 0.0001) were significantly shorter in group A. Group B showed a greater reliance on 3D mapping (7.2% vs. 89.5%, *p* < 0.0001), a shorter fluoroscopy time (23.5 ± 13.8 vs. 21.5 ± 15.1, *p* < 0.0001) and a lower dose area product (2,377.0 [1,186.0, 4,700.0] vs. 1,724.0 [768.0, 3,362.0], *p* < 0.0001). For further details, refer to Supplementary Table S3.

Both groups experienced similar rates of total procedural complications (7.6% vs. 10.1%, *p* = 0.07), with comparable distributions of major adverse cerebral and cardiovascular events (MACCE) (*p* = 0.06) and major complications (*p* = 0.7). Minor complications were more common in group B (5.9% vs. 9.1%, *p* < 0.01), particularly pericardial effusion or pericarditis (0.6% vs. 2.3%, *p* < 0.001) and groin complications treated conservatively (2.6% vs. 4.9%, *p* < 0.05). There was no significant difference in total posthospital complications (4.7% vs. 6.4%, *p* = 0.14), but group B experienced more posthospital major complications (1.3% vs. 3.5%, *p* < 0.01), including higher rates of pulmonary vein stenosis (0.6% vs. 1.9%, *p* < 0.01), severe bleeding (0.2% vs. 0.8%, *p* < 0.05), and pericardial effusions requiring intervention (0.1% vs. 1.9%, *p* < 0.01). Detailed complication data can be found in Supplementary Tables S1, S2.

### Follow-up-data

The median follow-up was 434 (375, 517) days, with group A having a significantly shorter follow-up time compared to group B (422 days vs. 482 days, *p* < 0.0001). Atrial arrhythmia recurrence occurred in 47.9% of patients, with 45.0% in group A and 56.9% in group B (*p* < 0.0001). Repeat ablation was required in 7.9% of group A patients and 22.3% of group B patients (*p* < 0.001). Group A also had fewer cardioversions (9.2% vs. 14.6%, *p* < 0.001) and rehospitalizations (26% vs. 46.7%, *p* < 0.0001) compared to group B. Furthermore, patient satisfaction was significantly higher in group A (*p* < 0.0001). Additional details are provided in Table 2.

**Table 2:** Follow-up data.

Follow-up data	Total (*n* = 2,110)	Group A (*n* = 1,590)	Group B (*n* = 520)	*p*-value
Follow-up performed (%)	2,110 (100)	1,590 (100)	520 (100)	
Days since intervention, days	434.0 (375.0, 517.0)	422.0 (372.0, 512.0)	482.0 (406.5, 527.0)	<0.0001
Arrhythmia recurrence (%)	1,002/2,091 (47.9)	709/1,576 (45.0)	293/515 (56.9)	<0.0001
EHRA				<0.0001
I/no (%)	1,533/2,064 (74.3)	1,186/1,554 (76.3)	347/510 (68.0)	
II/mild (%)	388/2,064 (18.8)	274/1,554 (17.6)	114/510 (22.4)	
III/severe (%)	122/2,064 (5.9)	81/1,554 (5.2)	41/510 (8.0)	
IV/disabling (%)	21/2,064 (1.0)	13/1,554 (0.8)	8/510 (1.6)	
Repeat ablations (%)	240/2,097 (11.4)	125/1,581 (7.9)	115/516 (22.3)	<0.0001
Cardioversions (%)	220/2,093 (10.5)	145/1,578 (9.2)	75/515 (14.6)	<0.001
Rehospitalisation since discharge (%)	650/2,091 (31.1)	409/1,575 (26.0)	241/516 (46.7)	<0.0001
Causes of rehospitalization				<0.05
Atrial fibrillation (%)	319/637 (50.1)	187/401 (46.6)	132/236 (55.9)	
Other CV cause (%)	107/637 (16.8)	68/401 (17.0)	39/236 (16.5)	
Non-CV cause (%)	211/637 (33.1)	146/401 (36.4)	65/236 (27.5)	
Patient satisfaction				<0.0001
Overall successful (%)	1,080/1,672 (64.6)	803/1,156 (69.5)	277/516 (53.7)	
Partially successful (%)	309/1,672 (18.5)	192/1,156 (16.6)	117/516 (22.7)	
Not successful (%)	197/1,672 (11.8)	105/1,156 (9.1)	92/516 (17.8)	
Not determinable (%)	19/1,672 (1.1)	8/1,156 (0.7)	11/516 (2.1)	
Unknown (%)	67/1,672 (4.0)	48/1,156 (4.2)	19/516 (3.7)	
Question not asked (%)	0/1,672 (0.0)	0/1,156 (0.0)	0/516 (0.0)	
Feeling safe (%)	1,599/1,644 (97.3)	1,112/1,138 (97.7)	487/506 (96.2)	0.09
Return to the same clinic (%)	1,589/1,637 (97.1)	1,104/1,132 (97.5)	485/505 (96.0)	0.10

*n* (%), Mean ± SD, or Median (Quartiles) as appropriate according to the test of normal distribution. CV, cardiovascular.

### Quality-of-life data

At baseline, both groups reported significant impairments in HRQoL, with 89.7% of group A and 92.9% of group B exhibiting an EHRA symptom score ≥ II (*p* < 0.0001). However, there were several baseline differences in EQ-5D-3L scores. Group B demonstrated worse outcomes in mobility (*p* < 0.05) and usual activities (*p* < 0.01), whereas group A reported worse pain/discomfort (*p* < 0.0001). Detailed results are shown in Tables 1, 3.

**Table 3:** Preprocedural quality of life data.

Preprocedural quality of life data	Total (*n* = 2,110)	Group A (*n* = 1,590)	Group B (*n* = 520)	*p*-value
Mobility				<0.05
Patient has no problems (%)	1,899 (90.0)	1,444 (90.8)	455 (87.5)	
Patient has some problems (%)	210 (10.0)	146 (9.2)	64 (12.5)	
Patient is bedridden (%)	1 (0.0)	0 (0.0)	1 (0.2)	
Self-care				0.44
Patient has no problems (%)	2,066 (97.9)	1,559 (98.1)	507 (97.5)	
Patient has some problems (%)	41 (1.9)	30 (1.9)	11 (2.1)	
Patient is unable (%)	3 (0.1)	1 (0.1)	2 (0.4)	
Usual activities				<0.01
Patient has no problems (%)	1,763 (83.6)	1,350 (84.9)	413 (79.4)	
Patient has some problems (%)	341 (16.2)	237 (14.9)	104 (20.0)	
Patient is unable (%)	6 (0.3)	3 (0.2)	3 (0.6)	
Pain/Discomfort				<0.0001
No pain or discomfort (%)	1,243 (58.9)	888 (55.8)	355 (68.3)	
Moderate pain or discomfort (%)	838 (39.7)	678 (42.6)	160 (30.8)	
Extreme pain or discomfort (%)	29 (1.4)	24 (1.5)	5 (1.0)	
Anxiety/Depression				0.32
Neither anxious or depressed (%)	1,599 (75.8)	1,214 (76.4)	385 (74.0)	
Moderatly anxious or depressed (%)	485 (23)	354 (22.3)	131 (25.2)	
Extremely anxious or depressed (%)	26 (1.2)	22 (1.4)	4 (0.8)	
General physical condition				0.35
GPC during the past 12 months: better (%)	212 (10.0)	155 (9.7)	57 (11.0)	
GPC during the past 12 months: same (%)	1,342 (63.6)	1,030 (64.8)	312 (60.0)	
GPC during the past 12 months: worse (%)	556 (26.4)	405 (25.5)	151 (29.0)	
EuroQol 5D-Score—Greiner 2005 (%)	0.9 ± 0.1	0.9 ± 0.1	0.9 ± 0.1	<0.001

*n* (%), Mean ± SD, or Median (Quartiles) as appropriate according to the test of normal distribution. GPC, general physical condition.

At follow-up, both groups showed improvements in EHRA symptom score and EQ-5D-3L. However, differences between the groups persisted. Group A exhibited a lower proportion of patients with an EHRA symptom score ≥ II (23.7% vs. 32%, *p* < 0.0001). In addition, group A demonstrated significantly better outcomes across multiple EQ-5D-3L dimensions, including mobility (*p* < 0.001), usual activities (*p* < 0.0001), pain/discomfort (*p* < 0.05), anxiety/depression (*p* < 0.01), and GPC (*p* < 0.001). Further details are provided in Tables 2, 4.

**Table 4:** Quality of life data at follow-up.

Postprocedural quality of life data	Total (*n* = 2,110)	Group A (*n* = 1,590)	Group B (*n* = 520)	*p*-value
Mobility				<0.001
Patient has no problems (%)	1,865 (88.4)	1,428 (89.8)	437 (84.0)	
Patient has some problems (%)	242 (11.5)	160 (10.1)	82 (15.8)	
Patient is bedridden (%)	3 (0.1)	2 (0.1)	1 (0.2)	
Self-care				0.06
Patient has no problems (%)	2,047 (97.0)	1,549 (97.4)	498 (95.8)	
Patient has some problems (%)	60 (2.8)	38 (2.4)	22 (4.2)	
Patient is unable (%)	3 (0.1)	3 (0.2)	0 (0.0)	
Usual activities				<0.0001
Patient has no problems (%)	1,786 (84.6)	1,376 (86.5)	410 (78.8)	
Patient has some problems (%)	304 (14.4)	202 (12.7)	102 (19.6)	
Patient is unable (%)	20 (0.9)	12 (0.8)	8 (1.5)	
Pain/Discomfort				<0.05
No pain or discomfort (%)	1,573 (74.5)	1,201 (75.5)	372 (71.5)	
Moderate pain or discomfort (%)	452 (21.4)	336 (21.1)	116 (22.3)	
Extreme pain or discomfort (%)	85 (4.0)	53 (3.3)	32 (6.2)	
Anxiety/Depression				<0.01
Neither anxious or depressed (%)	1,723 (81.7)	1,321 (83.1)	402 (77.3)	
Moderatly anxious or depressed (%)	341 (16.2)	239 (15.0)	102 (19.6)	
Extremely anxious or depressed (%)	46 (2.2)	30 (1.9)	16 (3.1)	
General physical condition				<0.001
GPC during the past 12 months: better (%)	1,428 (67.7)	1,110 (69.8)	318 (61.2)	
GPC during the past 12 months: same (%)	565 (26.8)	401 (25.2)	164 (31.5)	
GPC during the past 12 months: worse (%)	117 (5.5)	79 (5.0)	38 (7.3)	
EuroQol 5D-Score—Greiner 2,005 (%)	0.9 ± 0.2	0.9 ± 0.2	0.9 ± 0.2	<0.001

*n* (%), Mean ± SD, or Median (Quartiles) as appropriate according to the test of normal distribution. GPC, general physical condition.

When assessing changes over time, the magnitude of HRQoL improvement from baseline to follow-up differed significantly between groups. Patients in the CBA group demonstrated greater improvements in pain/discomfort, GPC, EQ-VAS, and the overall EuroQol 5D-Score compared to the RFA group (all *p* < 0.001). Detailed results are illustrated in Figure 3.

**Figure 3:**
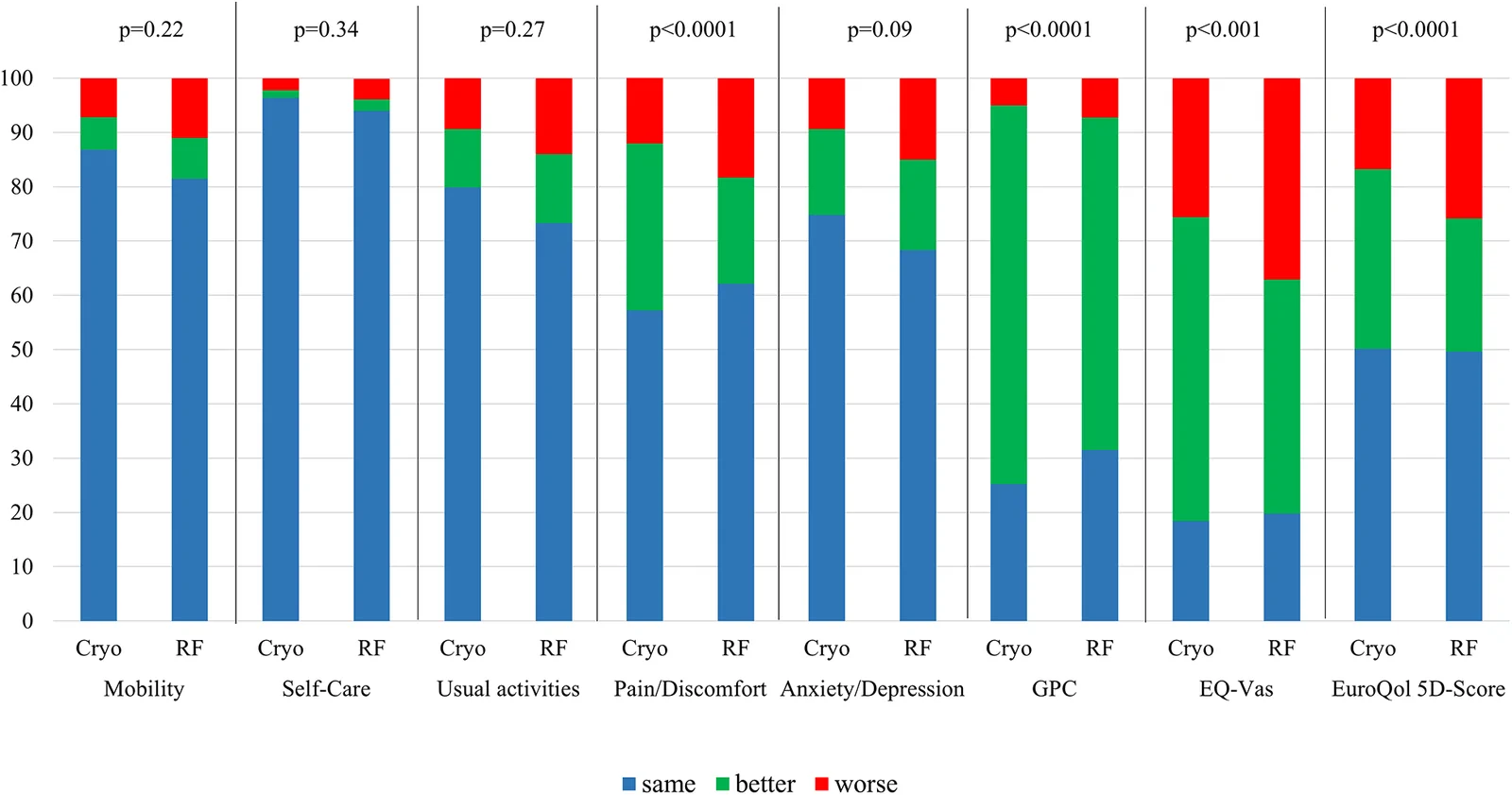
Comparison of HRQoL improvements in CBA and RFA. Bar chart showing the distribution of changes in HRQoL across various dimensions, including mobility, self-care, usual activities, pain/discomfort, anxiety/depression, general physical condition (GPC), EQ-VAS, and EuroQoL 5D score. Each category represents the percentage of patients in the CBA (Cryo) and RFA (RF) groups reporting “same,” “better,” or “worse” HRQoL compared to baseline. Green indicates improvement, blue represents no change, and red indicates worsening. *P*-values shown represent unadjusted between-group comparisons. After multivariable adjustment, significant associations with CBA remained for improvement in pain/discomfort, EQ-VAS, and the EuroQol 5D-Score.

In multivariable analyses, group A remained associated with a higher likelihood of improvement in pain/discomfort (aOR 1.62, 95% CI 1.26–2.08; *p* < 0.001), EQ-VAS (aOR 1.60, 95% CI 1.27–2.02; *p* < 0.0001), and the EuroQol 5D-Score (aOR 1.39, 95% CI 1.10–1.76; *p* = 0.007). No significant associations were observed for mobility, self-care, usual activities, anxiety/depression, or GPC. In the analysis of continuous changes, the adjusted least-squares mean difference between CBA and RFA was 2.73 points for EQ-VAS (95% CI 1.35–4.10) and 0.04 points for the EuroQol 5D-Score (95% CI 0.02–0.05).

In subgroup analyses stratified by AF type, associations between ablation modality and HRQoL change were assessed separately in patients with paroxysmal and persistent AF. Among patients with paroxysmal AF, significant associations were observed for pain/discomfort (*p* = 0.0001), anxiety/depression (*p* = 0.045), EQ-VAS (*p* = 0.002), and the EuroQol 5D-Score (*p* = 0.0002). In patients with persistent AF, a significant association was observed for EQ-VAS (*p* = 0.007), whereas no significant associations were observed for the remaining HRQoL outcomes (Supplementary Figure S1).

## Discussion

This analysis provides one of the largest real-world comparisons of CBA and RFA for the treatment of symptomatic AF with a focus on PRO. Moreover, the distinctiveness of these results stems from the FREEZE Cohort Trial being a multicenter, multinational study that captured real-world data from centers experienced in the designated ablation techniques. In this clustered cohort design, centers with expertise in either RFA or CBA were pre-assigned to their preferred technique before the trial commenced.

The main findings of this analysis are: Firstly, both CBA and RFA are safe and effective modalities for the treatment of symptomatic AF patients leading to an improvement in HRQoL compared to baseline. Second, after multivariable adjustment, CBA remained associated with greater improvements in pain/discomfort, EQ-VAS, and the EuroQol 5D-Score compared with RFA.

Regarding the effectiveness of the two ablation modalities, it is noteworthy that the AF recurrence rates were different between the two groups. While presenting a noteworthy AF recurrence of 47.9% with a median follow-up of 434 days as well as a high overall satisfaction level of 64.6%, group B had a significantly higher rate of atrial arrhythmia recurrence compared to group A, as well as a higher requirement for repeat ablations. These findings are inconsistent with previous trials comparing RFA and CBA in AF patients. In the FIRE AND ICE trial, which assessed CBA and RFA in paroxysmal AF patients, arrhythmia recurrence was similar in both groups (11). However, a recent meta-analysis indicates a significant reduction in the incidence of AF recurrence with CBA compared to RFA, without focusing on a specific subtype of AF (12).

The observed differences in clinical outcomes may partly reflect differences in baseline characteristics and follow-up duration between the two groups. Group B patients were generally older, had a higher prevalence of persistent AF, and experienced a longer follow-up compared to group A. Additionally, they had higher EHRA symptom scores and a greater burden of comorbidities, including hypertension, coronary artery disease, and obesity. These factors are known to contribute to poorer outcomes after AF ablation due to their association with increased atrial remodeling and the development of a more complex atrial substrate, which can complicate achieving durable rhythm control and increase the likelihood of arrhythmia recurrence, even with adequate PVI (1, 13).

Consistent with the higher observed recurrence rate, repeat ablations, cardioversions, and rehospitalizations were also more frequent in the RFA group. While these findings indicate differences in the clinical course following ablation, differences in baseline characteristics and follow-up duration should be considered in their interpretation.

It is noteworthy that total complications were similarly distributed between both groups, but minor complications were more prevalent in the RFA group. This difference was primarily driven by a higher incidence of groin complications and pericardial effusions in the RFA group. These findings are consistent with a previously published meta-analysis by Fortuni et al., which compared CBA and RFA as first-line procedures for AF (12). The higher rate of groin complications in RFA procedures, which typically use a smaller sheath diameter (8.5F), compared to CBA's larger 15F sheath, may be linked to the use of a two-sheath system during RFA, which involves both a radiofrequency catheter and a separate circular mapping catheter in the left atrium (11, 14). This also corresponds with the greater use of both total and left atrial catheters in the RFA group, as observed in the present study. Beyond the risk related to transseptal puncture, the higher incidence of pericardial effusion in the RFA group may result from tissue overheating and steam pops, which can cause myocardial perforation and life-threatening tamponade (12). However, it is important to note that CBA was associated with a significantly higher rate of transient phrenic nerve palsy compared to RFA.

At baseline, both the CBA and RFA groups reported significant impairments in HRQoL with the majority of patients in both groups exhibiting an EHRA symptom score ≥ II, indicating moderate to severe symptoms. The RFA group had worse scores for mobility and usual activities, suggesting that patients in this group experienced greater physical limitations. Conversely, group A reported more severe pain/discomfort highlighting the different symptom profiles between the two groups at baseline. At follow-up, both groups demonstrated improvements in their EHRA symptom scores and EQ-5D-3L scores, suggesting that both ablation techniques contributed to symptom relief and HRQoL improvement. These findings are in line with previously published papers reporting significant but similar HRQoL improvement in symptomatic AF patients undergoing either CBA or RFA (7, 15, 16).

Despite improvements in both groups, differences in HRQoL persisted at follow-up. Given the baseline imbalances between the groups, unadjusted comparisons should therefore be interpreted with caution.

It is important to note that group A showed a significantly greater reduction in EHRA symptom scores ≥ II at follow-up, indicating more substantial symptom improvement compared to group B. Additionally, greater improvements in pain/discomfort, GPC, EQ-VAS, and the EuroQoL 5D score were observed in the CBA group compared with the RFA group. However, given the differences in baseline characteristics and follow-up duration between the groups, these findings should be interpreted as associations and not as evidence of superiority of CBA over RFA. Notably, after multivariable adjustment, CBA remained associated with greater improvement in pain/discomfort, EQ-VAS, and the EuroQol 5D-Score, whereas no significant association was observed for the other HRQoL domains. These findings suggest that the observed differences in selected HRQoL measures were not entirely explained by the measured baseline differences between the treatment groups. However, residual confounding inherent to the observational study design cannot be excluded.

In subgroup analyses stratified by AF type, associations between ablation modality and several HRQoL outcomes were observed predominantly in patients with paroxysmal AF, including pain/discomfort, anxiety/depression, EQ-VAS, and the EuroQol 5D-Score, whereas in patients with persistent AF, a significant association was observed only for EQ-VAS. This finding is particularly relevant given the substantial difference in AF type between the treatment groups and suggests that AF type may contribute to the observed heterogeneity in HRQoL outcomes. At the same time, the persistence of differences in several HRQoL measures within the paroxysmal AF subgroup indicates that the overall findings cannot be attributed solely to the higher prevalence of persistent AF in the RFA group. Given the exploratory nature of the subgroup analysis, however, these findings should be interpreted cautiously.

In contrast, prior research comparing HRQoL outcomes between CBA and RFA has not shown significant differences, with most data indicating similar HRQoL improvements, though this remains limited (16, 17). More recently, Matteucci et al. compared PRO across different ablation energy sources, including conventional RF, high-power short-duration RF, CBA, and pulsed-field ablation (PFA), and reported significant differences in perceived QoL and symptom improvement between energy sources, with the greatest improvements observed following PFA. These findings further suggest that the choice of ablation energy source may influence specific PRO and highlight the need to incorporate PROs when evaluating contemporary AF ablation strategies (18).

The discrepancy between our findings and previous studies may reflect differences in patient populations, study design, baseline characteristics, and clinical outcomes. Nevertheless, the cluster cohort design represents a valuable study approach with high real-world applicability. By reflecting routine clinical practice in experienced centers with predefined ablation strategies, it may reduce treatment-selection bias at the individual patient level. While randomized controlled trials offer high internal validity, their strict protocols and selected patient populations may limit generalizability. In contrast, cluster cohort studies provide complementary evidence by capturing practice-based outcomes across diverse healthcare settings.

In this sub-analysis, HRQoL was assessed using the EQ-5D-3L instrument, which evaluates five key dimensions of health: mobility, self-care, usual activities, pain/discomfort, and anxiety/depression. Both CBA and RFA led to significant improvements from baseline across all five EQ-5D-3L domains, with comparable gains in mobility, self-care, usual activities, and anxiety/depression between the two modalities. Notably, greater improvement in pain/discomfort was observed in the CBA group, and this association remained significant after multivariable adjustment, suggesting a more pronounced reduction in pain-related symptom burden compared with RFA. These patient-reported improvements underscore that HRQoL, as a PRO measure, provides a reliable and complementary endpoint alongside traditional clinical metrics such as atrial arrhythmia recurrence, arrhythmia burden or time to recurrence, which do not fully capture the patient's daily well-being. Indeed, beyond prognostic considerations or rhythm-focused outcomes, quality of life itself emerges as a highly relevant clinical endpoint in AF management, directly reflecting the tangible benefits of therapy from the patient's perspective. Ultimately, our findings reaffirm that when PVI is achieved, catheter ablation—regardless of modality—yields substantial HRQoL gains, highlighting that successful ablation translates into meaningful improvements in patient quality of life.

### Limitations

This study has several limitations that should be considered when interpreting the results. First, it represents a sub-analysis of the FREEZE Cohort Study and was not specifically designed to assess HRQoL outcomes, introducing the potential for unmeasured bias. Second, the cluster-based study design, in which participating centers predefined their preferred ablation modality, may have resulted in selection bias and confounding by indication. Consequently, the RFA group comprised older patients with more advanced AF and a higher burden of comorbidities. Although multivariable adjustment was performed to account for measured differences between the treatment groups, residual confounding due to unmeasured or incompletely captured factors cannot be excluded. Therefore, the observed associations should not be interpreted as evidence of a causal treatment effect or superiority of either ablation modality. Third, follow-up duration differed between groups (422 days for CBA vs. 482 days for RFA), which may have affected recurrence rates and long-term outcomes, potentially leading to an underestimation of late events in the CBA group. Furthermore, while HRQoL was measured using well-established tools such as the EQ-5D-3L and the EHRA symptom score, the subjective nature of HRQoL assessments means that PRO may be influenced by individual expectations or reporting biases, particularly in a non-blinded study design. Finally, this study did not investigate the impact of different procedural factors on HRQoL outcomes, which could provide additional insights into the relationship between procedural factors and patient-reported outcomes.

## Conclusion

In this large, prospective, real-world cohort, both CBA and RFA led to significant improvements in HRQoL in patients with symptomatic AF. After multivariable adjustment, CBA remained associated with greater improvements in pain/discomfort, EQ-VAS, and the EuroQol 5D-Score, suggesting differences in selected PRO between ablation modalities. Importantly, our findings underscore that catheter ablation—regardless of technique—can substantially improve HRQoL when PVI is achieved. HRQoL emerges as a reliable and meaningful endpoint, complementing traditional clinical outcomes and reinforcing the patient-centered value of AF ablation in routine care.

## Data Availability

The raw data supporting the conclusions of this article may be made available upon reasonable scientific request, provided that data sharing is consistent with the informed consent of the study participants.

## FREEZE Cohort Study Investigators

Electrophysiology, University Hospital Virgen de la Arrixaca, El Palmar, Spain: A. Garcia Alberola. Klinik für Kardiologie und intern. Intensivmedizin, Klinikum Bielefeld Mitte, Bielefeld, Germany: C. Stellbrink. Klinik für Innere Med. III, Kardiologie, Angiologie u. Pneumologie, Universitätsklinikum Heidelberg, Heidelberg, Germany: D. Thomas, H. A. Katus. Heidelberger Zentrum für Herzrhythmusstörungen (HCR), Medizinische Universitätsklinik Heidelberg, Heidelberg, Germany: D. Thomas. Deutsches Institut für Herzkreislaufforschung, Standort Heidelberg/Mannheim, Heidelberg, Germany: D. Thomas. Medizinische Klinik III—CCB, Agaplesion Markus Krankenhaus, Frankfurt am Main, Germany: K. R. J. Chun. Medizinische Klinik IV, Klinikum Hanau GmbH, Hanau, Germany: G. Groschup. Klinik für Innere Medizin—Kardiologie, Deutsches Herzzentrum Berlin, Berlin, Germany: J.-H. Gerds-Li. Medizinische Klinik und Poliklinik II, Universitätsklinikum Bonn, Bonn, Germany: J. W. Schrickel. Klinik für Kardiologie/Elektrophysiologie, Universitäres Herz- und Gefäßzentrum Hamburg GmbH, Hamburg, Germany: A. Metzner. Klinik für Rhythmologie, Universitätsklinikum Schleswig-Holstein, Lübeck, Germany: R. R. Tilz. Cardiology, Netcare Sunninghill Hospital, Sandton, South Africa: A. Stanley. Cardiology, Milpark Hospital, Parktown west, South Africa: I. W. P. Obel. Cardiology, Panorama Medi-Clinic, Cape Town, South Africa: R. R. Gopal. Gemeinschaftspraxis Kardiologie und Pneumologie, Mönchengladbach, Germany: L. Lickfett. Department of Cardiology and Internal Diseases, Medical University of Gdańsk, Gdańsk, Poland: A. Lubinski. Klinik für Innere Medizin II, Westpfalz-Klinikum GmbH, Kaiserslautern, Germany: B. Schumacher. Cardiology, Mission Hospital, Asheville, United States: J. J. Souza. Kardiologie und internistische Intensivmedizin, Kepler Universitätsklinikum, Linz, Austria: C. Steinwender. Klinik für Innere Medizin 1—Kardiologie, Klinikum Frankfurt Höchst GmbH, Frankfurt am Main, Germany: H. U. Hink. Abteilung Kardiologie, Klinikum Siloah, Hannover, Germany: A. Franke. Stiftung Institut für Herzinfarktforschung, Ludwigshafen, Germany: T. Ouarrak. Medical School/Regiomed GmbH, Coburg, Germany: J. Brachmann.
