# Supplementary material for: Quality of life after cryoballoon vs. radiofrequency ablation in atrial fibrillation: a subanalysis of the FREEZE cohort study
Source: Front Cardiovasc Med. 2026 Oct 9;13:1877743. doi: 10.3389/fcvm.2026.1877743 (PMC13690627; doi:10.3389/fcvm.2026.1877743)
Supplement: Supplementary file 1 [file Supplementaryfile1.docx]

**Supplement Figure 1: Changes in HRQoL following CBA and RFA stratified by AF type.**

Bar charts showing the distribution of changes in HRQoL from baseline to follow-up in patients with (A) paroxysmal AF and (B) persistent AF. Changes in mobility, self-care, usual activities, pain/discomfort, anxiety/depression, EQ-VAS, and the EQ-5D index are categorized as “same,” “better,” or “worse” compared with baseline. Blue indicates no change, green indicates improvement, and red indicates worsening. P-values shown represent unadjusted between-group comparisons of CBA (Cryo) and RFA (RF).
