# Supplementary material for: Quality of life after cryoballoon vs. radiofrequency ablation in atrial fibrillation: a subanalysis of the FREEZE cohort study
Source: Front Cardiovasc Med. 2026 Oct 9;13:1877743. doi: 10.3389/fcvm.2026.1877743 (PMC13690627; doi:10.3389/fcvm.2026.1877743)
Supplement: Supplementary file 2 [file Table1.docx]

|  | **Total (n=2110)** | **Group A (n=1590)** | **Group B (n=520)** | **p-value** |
| --- | --- | --- | --- | --- |
| **Procedural complications (%)** | 171/2083 (8.2) | 119/1569 (7.6) | 52/514 (10.1) | 0.07 |
| **Hospital MACCE** | 5/2108 (0.2) | 3/1590 (0.2) | 2/518 (0.4) | 0.42 |
| **Death (%)** | 0/2109 (0.0) | 0/1590 (0.0) | 0/519 (0.0) | - |
| **Myocardial infarction (%)** | 0/2109 (0.0) | 0/1590 (0.0) | 0/519 (0.0) | - |
| **Stroke (%)** | 5/2108 (0.2) | 3/1590 (0.2) | 2/518 (0.4) | - |
| **Major complications (%)** | 40/2061 (1.9) | 30/1547 (1.9) | 10/514 (1.9) | 0.99 |
| **Persistent phrenic nerve palsy (%)** | 15/2085 (0.7) | 15/1567 (1.0) | 0/518 (0.0) | <0.05 |
| **Tamponade or pericardial effusion treated by drainage or surgery (%)** | 8/2108 (0.4) | 5/1590 (0.3) | 3/518 (0.6) | 0.39 |
| **Severe bleeding (%)** | 6/2108 (0.3) | 3/1590 (0.2) | 3/518 (0.6) | 0.15 |
| **Resuscitation (%)** | 0/2108 (0.0) | 0/1590 (0.0) | 0/518 (0.0) | - |
| **TIA < 24h (%)** | 3/2108 (0.1) | 1/1590 (0.1) | 2/518 (0.4) | 0.09 |
| **Pulmonary embolism (%)** | 0/2108 (0.0) | 0/1590 (0.0) | 0/518 (0.0) | - |
| **Hemato/pneumothorax (%)** | 0/2108 (0.0) | 0/1590 (0.0) | 0/518 (0.0) | - |
| **Groin complications treated interventionally or with surgery (%)** | 9/2083 (0.4) | 7/1569 (0.4) | 2/514 (0.4) | 0.86 |
| **Minor complications (%)** | 138/2068 (6.7) | 91/1554 (5.9) | 47/514 (9.1) | < 0.01 |
| **Transient phrenic nerve palsy (%)** | 23/2093 (1.1) | 23/1575 (1.5) | 0/518 (0.0) | < 0.001 |
| **Pericardial effusion or pericarditis (%)** | 21/2108 (1.0) | 9/1590 (0.6) | 12/518 (2.3) | < 0.001 |
| **Groin complications treated conservatively (%)** | 66/2083 (3.2) | 41/1569 (2.6) | 25/514 (4.9) | <0.05 |
| **Cardiac complication (arrhythmia, decompensation) (%)** | 2/2108 (0.1) | 1/1590 (0.1) | 1/518 (0.2) | 0.40 |
| **Moderate bleeding (%)** | 9/2108 (0.4) | 7/1590 (0.4) | 2/517 (0.4) | 0.87 |
| **Systemic infection (%)** | 3/2108 (0.1) | 1/1590 (0.1) | 2/518 (0.4) | 0.09 |
| **Esophageal lesion (%)** | 0/2108 (0.0) | 0/1590 (0.0) | 0/518 (0.0) | - |
| **Bronchial complication (%)** | 6/2108 (0.3) | 5/1590 (0.3) | 1/518 (0.2) | 0.65 |
| **Haematuria (%)** | 2/2108 (0.1) | 1/1590 (0.1) | 1/518 (0.2) | 0.4 |
| **Allergic reaction (%)** | 2/2108 (0.1) | 0/1590 (0.0) | 2/518 (0.4) | <0.05 |
| **Other complications (%)** | 8/2108 (0.4) | 5/1590 (0.3) | 3/518 (0.6) | 0.39 |
