# Supplementary material for: Quality of life after cryoballoon vs. radiofrequency ablation in atrial fibrillation: a subanalysis of the FREEZE cohort study
Source: Front Cardiovasc Med. 2026 Oct 9;13:1877743. doi: 10.3389/fcvm.2026.1877743 (PMC13690627; doi:10.3389/fcvm.2026.1877743)
Supplement: Supplementary file 3 [file Table2.docx]

|  | **Total (n=2,110)** | **Group A (n=1590)** | **Group B (n=520)** | **p-value** |
| --- | --- | --- | --- | --- |
| **Posthospital total complications (%)** | 107/2083 (5.1) | 74/1566 (4.7) | 33/517 (6.4) | 0.14 |
| **Posthospital MACCE** | 9/2098 (0.4) | 6/1580 (0.4) | 3/518 (0.6) | 0.55 |
| **Death (%)** | 0/2110 (0.0) | 0/1590 (0.0) | 0/520 (0.0) | - |
| **Causes of death** | 0/0 (0.0) | 0/0 (0.0) | 0/0 (0.0) | - |
| **Cardiovascular (%)** | 0/0 (0.0) | 0/0 (0.0) | 0/0 (0.0) | - |
| **Non-Cardiovascular (%)** | 0/0 (0.0) | 0/0 (0.0) | 0/0 (0.0) | - |
| **Unknown (%)** | 0/0 (0.0) | 0/0 (0.0) | 0/0 (0.0) | - |
| **Posthospital myocardial infarction (%)** | 2/2101 (0.1) | 1/1582 (0.1) | 1/519 (0.2) | 0.41 |
| **Posthospital Stroke (%)** | 7/2101 (0.3) | 5/1583 (0.3) | 2/518 (0.4) | 0.81 |
| **Posthospital major complications (%)** | 38/2085 (1.8) | 20/1567 (1.3) | 18/518 (3.5) | < 0.01 |
| **TIA (%)** | 1/2099 (0.0) | 1/1582 (0.1) | 0/517 (0.0) | 0.57 |
| **PV Stenosis (%)** | 19/2100 (0.9) | 9/1581 (0.6) | 10/519 (1.9) | < 0.01 |
| **Atrio-esophageal fistula (%)** | 0/2101 (0.0) | 0/1582 (0.0) | 0/519 (0.0) | - |
| **Persistent phrenic nerve palsy (%)** | 8/2103 (0.4) | 7/1584 (0.4) | 1/519 (0.2) | 0.42 |
| **Severe bleeding (%)** | 7/2104 (0.3) | 3/1586 (0.2) | 4/518 (0.8) | < 0.05 |
| **Pericardial effusion treated interventionally or with surgery (%)** | 4/2103 (0.2) | 1/1582 (0.1) | 3/519 (0.6) | < 0.05 |
| **Pulmonary embolism (%)** | 2/2101 (0.1) | 2/1582 (0.1) | 0/519 (0.0) | 0.42 |
| **Posthospital minor complications (%)** | 85/2093 (4.1) | 59/1576 (3.7) | 26/517 (5.0) | 0.20 |
| **Moderate bleeding (%)** | 17/2104 (0.8) | 12/1586 (0.8) | 5/518 (1.0) | 0.65 |
| **Pericardial effusion treated conservatively (%)** | 8/2103 (0.4) | 6/1584 (0.4) | 2/519 (0.4) | 0.98 |
| **Syncopes (%)** | 11/2101 (0.5) | 9/1582 (0.6) | 2/519 (0.4) | 0.62 |
| **Phlebothrombosis (%)** | 2/2101 (0.1) | 2/1582 (0.1) | 0/519 (0.0) | 0.42 |
| **Inguinal problems (%)** | 52/2102 (2.5) | 33/1584 (2.1) | 19/518 (3.7) | < 0.05 |
