# Supplementary material for: Quality of life after cryoballoon vs. radiofrequency ablation in atrial fibrillation: a subanalysis of the FREEZE cohort study
Source: Front Cardiovasc Med. 2026 Oct 9;13:1877743. doi: 10.3389/fcvm.2026.1877743 (PMC13690627; doi:10.3389/fcvm.2026.1877743)
Supplement: Supplementary file 4 [file Table3.docx]

**Table 2: Procedural Results**

|  | **Total (n=2,110)** | **Group A (n=1590)** | **Group B (n=520)** | **p-value** |
| --- | --- | --- | --- | --- |
| **Total catheters (%)** | 3.0±0.9, n=2098 | 3.0±1.0, n=1584 | 2.9±1.1, n=514 | < 0.0001 |
| **LA catheters (%)** | 1.4±0.5, n=2089 | 1.2±0.4, n=1578 | 1.9±0.3, n=511 | < 0.0001 |
| **Electrophysiological Mapping (%)** | 2096/2110 (99.3) | 1580/1590 (99.4) | 516/520 (99.2) | 0.73 |
| **Conventional (%)** | 1516/2096 (72.3) | 1462/1580 (92.5) | 54/516 (10.5) | < 0.0001 |
| **3D Mapping (%)** | 576/2096 (27.5) | 114/1580 (7.2) | 562/516 (89.5) | < 0.0001 |
| **Fluoroscopy Time, min** | 23.0 ± 14.2 | 23.5 ± 13.8 | 21.5 ± 15.1 | < 0.0001 |
| **Dose area Procuct, cGy*cm²** | 2180.5 (1080.0, 4408.0), n=1990 | 2377.0 (1186.0, 4700.0), n=1493 | 1724.0 (768.0, 3362.0), n=497 | < 0.0001 |
| **LA Time, min** | 105.9±47.0, n=2038 | 95.4±37.9, n=1555 | 139.7±56.4, n=483 | < 0.0001 |
| **Procedure Time, min** | 141.9±57.5, n=2036 | 128.3±48.9, n=1547 | 184.8±61.5, n=489 | < 0.0001 |
| **ICE usage, %** | 655/2097 (31.2) | 596/1581 (37.7) | 59/516 (11.4) | < 0.0001 |
| **PV angiography, %** | 1555/2097 (74.2) | 1320/1581 (83.5) | 235/516 (45.5) | < 0.0001 |
| **Additional linear lesions (%)** | 220/2091 (10.5) | 186/1578 (11.8) | 34/513 (6.6) | < 0.001 |
| **Left atrial (%)** | 57/219 (26.0) | 53/185 (28.6) | 4/34 (11.8) | < 0.05 |
| **Right atrial (%)** | 12/219 (5.5) | 10/185 (5.4) | 2/34 (5.9) | 0.91 |
| **LA isthmus (%)** | 31/219 (14.2) | 20/185 (10.8) | 11/34 (32.4) | < 0.001 |
| **Roof line LA (%)** | 122/219 (55.7) | 104/185 (56.2) | 18/34 (52.9) | 0.72 |
| **RA isthmus (%)** | 76/219 (34.7) | 59/185 (31.9) | 17/34 (50.0) | < 0.05 |
| **Other (%)** | 16/219 (7.3) | 14/185 (7.6) | 2/34 (5.9) | 0.73 |
| **Isolation of all PV (%)** | 2006/2100 (95.5) | 1510/1583 (95.4) | 496/517 (95.9) | 0.60 |
| **LSPV (%)** | 2078/2100 (99.0) | 1566/1583 (98.9) | 512/517 (99.0) | 0.84 |
| **RSPV (%)** | 2079/2100 (99.0) | 1567/1583 (99.0) | 512/517 (99.0) | 0.93 |
| **LIPV (%)** | 2068/2100 (98.5) | 1557/1583 (98.4) | 511/517 (98.8) | 0.44 |
| **RIPV (%)** | 2053/2100 (97.8) | 1547/1583 (97.7) | 506/517 (97.9) | 0.84 |
| **Accessory (%)** | 73/146 (50.0) | 63/133 (47.4) | 10/13 (76.9) | < 0.05 |
| **Degree of difficulty** |  |  |  | 0.29 |
| **Simple (%)** | 934/2100 (44.5) | 713/1584 (45.0) | 221/516 (42.8) |  |
| **Moderately aggravated (%)** | 1040/2100 (49.5) | 781/1584 (49.3) | 259/516 (50.2) |  |
| **Severely aggravated (%)** | 126/2100 (6.0) | 90/1584 (5.7) | 36/516 (7.0) |  |

n (%), Mean ± SD, or Median (Quartiles) as appropriate according to the test of normal distribution. LA: left atrium, PV: pulmonary vein, LSPV: left superior pulmonary vein, RSPV: right superior pulmonary vein, LIPV: left inferior pulmonary vein, RIPV: right inferior pulmonary vein. ICE: intracardiac echocardiography.
